# THE PROMISE OF DIGITAL INCLUSION FOR OLDER ADULTS: BUILDING BRIDGES FOR UNDERSERVED OLDER ADULTS IN TWO STATES

**DOI:** 10.1093/geroni/igad104.1716

**Published:** 2023-12-21

**Authors:** Skye Leedahl, Kathleen Wilber, Namkee Choi

## Abstract

Digital inclusion refers to activities completed to ensure that all individuals, especially those who are underserved, have access to and are able to utilize technology and needed support. This includes affordable, robust internet service; devices; digital literacy training; technical support; and applications/online content (National Digital Inclusion Alliance, 2022). Recognizing digital exclusion among older adults as a major public health and social equity concern, particularly during the pandemic, two states (California and Rhode Island) partnered with their respective state units on aging to offer technological devices (e.g., iPads) and intergenerational support to underserved older adults who speak multiple languages across the states. The university-based programs worked with their local Areas Agencies on Aging (CA) and senior centers (RI) through the State units on Aging to recruit and support older participants. This symposium will describe findings from three research projects within these large, ongoing research initiatives and discuss some of the lessons learned for conducting this type of applied research. The first paper by Marnfeldt and colleagues describes factors associated with digital readiness among participants. The second paper by Leedahl and colleagues examines predictors of digital competence among those interested in the program. The third paper by Tsotsoros and colleagues examines the influence of technological growth on various social well-being measures.The fourth paper by Batista-Malat and colleagues describes the strengths and barriers in working with community partners when completing applied research.

